# Extracranial Vertebral Artery Aneurysm Presenting as a Chronic Cervical Mass Lesion

**DOI:** 10.1155/2010/938219

**Published:** 2010-04-06

**Authors:** Lampis C. Stavrinou, George Stranjalis, Pantelis C. Stavrinou, N. Bontozoglou, Damianos E. Sakas

**Affiliations:** ^1^Department of Neurosurgery, University of Athens Medical School, Evangelismos General Hospital, Pangrati, 116 32 Athens, Greece; ^2^Hellenic Center for Neurosurgical Research “Petros Kokkalis”, Greece; ^3^Department of Neurosurgery, University of Thessaloniki Medical School, AHEPA General Hospital, 54006 Thessaloniki, Greece

## Abstract

*Background*. Aneurysms of the extracranial vertebral artery are rare and can provide a diagnostic and therapeutic challenge. *Methods*. We reviewed the clinical history of a patient presenting with cervical radiculopathy, who harboured an extracranial vertebral artery aneurysm eroding the cervical spine. *Results*. CT Angiography and MR Angiography set the diagnosis, by revealing a left C5-C6 vertebral artery aneurysm with cervical root impingement. Bony reconstruction depicted enlargement of the C6 transverse foramen and a marked enlargement of the C6-C7 intravertebral foramen. The lesion was treated by intravascular proximal vertebral artery occlusion. *Conclusions*. Extracranial vertebral artery aneurysms require a high index of clinical suspicion. This is the first report of a vertebral artery pseudoaneurysm presenting with bony erosion, which supports a less minacious portrayal of vertebral artery aneurysms.

## 1. Case Report

A 61-year-old woman was referred to our department with a six-month history of vague neck pain with scapular radiation and rachialgia. She also complained of numbness of her left forearm, thumb, and the second and third digits. She was started on corticosteroids and anti-inflammatory drugs and underwent a cervical MRI. Radiological diagnosis was that of a left cervical plexus neurinoma.

**Figure 1:**
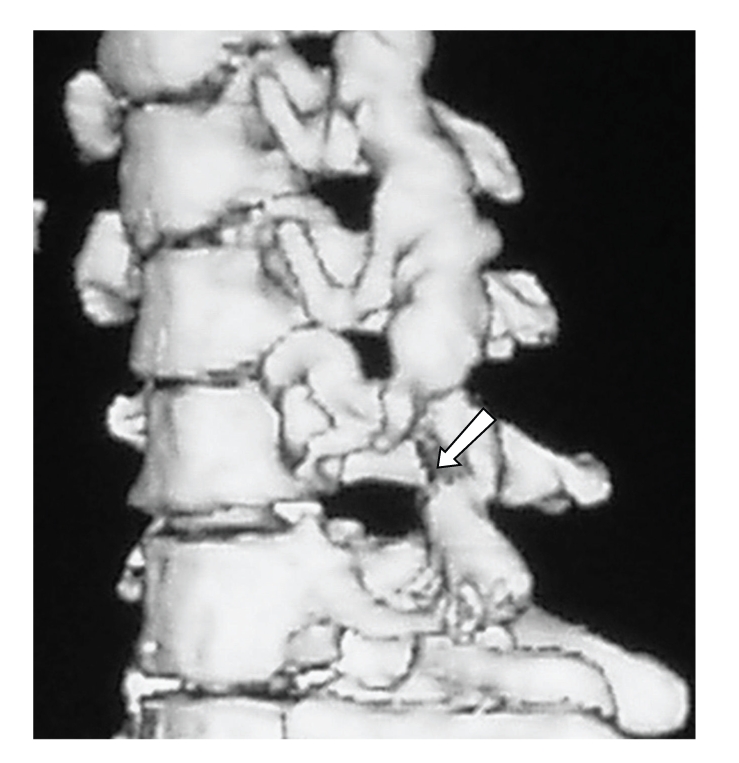
CT 3D reconstruction of the cervical spine shows erosion of the left intravertebral C6-C7 foramen (arrow).

**Figure 2:**
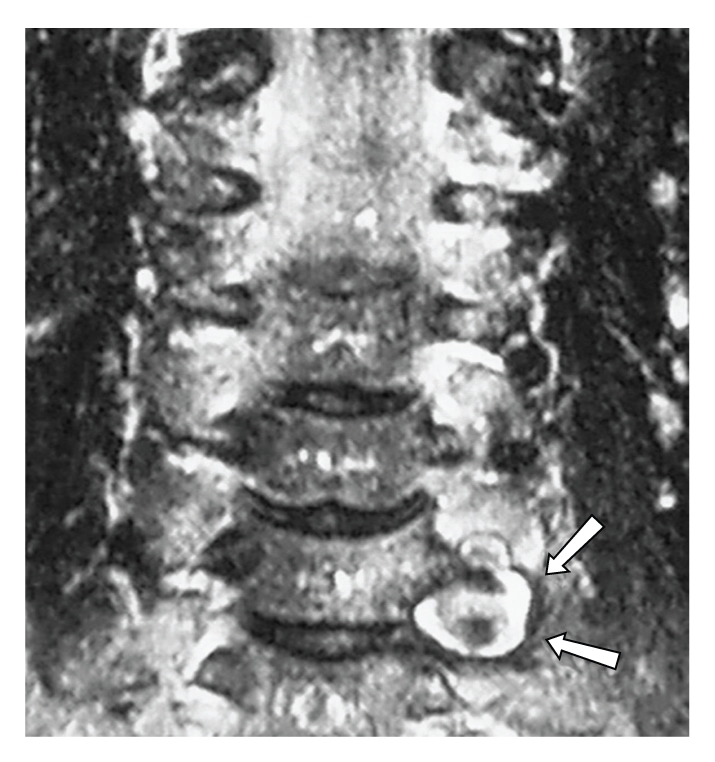
Coronal T2-weighted image of the cervical region; arrows point the aneurysm.

A few days later the patient's symptoms worsened. She started experiencing a burning sensation in her affected fingers and forearm and intense pain in her neck and left shoulder. At that point she consulted our department for a possible removal of her “neurinoma.” Under the impression that the Magnetic Resonance Imaging (MRI) performed a few weeks earlier at another center was technically unsatisfying and thus nondiagnostic, we performed an urgent CT scan of the cervical region with 3D reconstruction. The CT scan revealed an enlargement of the C6 transverse foramen and a marked enlargement of the C6-C7 intravertebral foramen **(** Figure 1 **)**. The latter was occupied by a 2 × 1,3 cm atractoid hyperdense lesion that protruded to the paraspinal tissues. Based on these findings, the possibility of a vascular lesion was raised, so a contrast-enhanced MRI/MR Angiography (MRA) scan of the cervical region and of the brain was performed. This confirmed the findings of the CT, again revealing an ovoid 2 cm × 1,6 cm lesion clearly adherent to the left vertebral artery, causing enlargement of the aforementioned foramina and enhancing homogenously after intravenous contrast administration **(** Figure 2 **)**. The images were suggestive of a vertebral artery pseudoaneurysm, while the affected vessel also appeared to have a double lumen right above the lesion that was attributed to a vertebral artery dissection. The brain MRI was unremarkable. The MRA also verified the absence of an intracranial aneurysm and confirmed the presence of adequate collateral blood supply by the contralateral vertebral artery in the face of a possible vertebral artery occlusion [1]. Having established adequate contralateral blood supply, the patient was treated with warfarine, and endovascular proximal occlusion of the left vertebral artery. 

A thorough review of the patient's history revealed no history of trauma. Fibromuscular dysplasia was considered in the differential diagnosis; however the absence of vertebral artery stenosis and the size of the aneurysm itself rendered this diagnosis rather unlikely. The patient, however, did mention having received chiropractic treatment for a “sore neck” two weeks prior to the onset of symptoms. 

## 2. Discussion

Isolated extracranial vertebral artery aneurysms are rare ailments, usually presenting after penetrating injuries and only rarely after blunt trauma. Schittek reviewed twenty-seven reports in the international literature, which described a total of 144 cases of verterbral artery injuries. These, included 22 pseudoaneurysms. Of those, only 8 were attributed to blunt trauma [1]. To the best of our knowledge, this is the first report of a vertebral artery pseudoaneurysm presenting with bony erosion and the second to be precipitated after chiropractic manipulation [2]. Pseudoaneurysms can vary from a few millimeters to a few centimeters, as was in our case, or even reach giant proportions. Small lesions may go unnoticed or be incidental findings; whereas larger ones are more probable to manifest with neurologic symptoms and deficits (radiculopathy, myelopathy or brain stem compression depending on the size and the location of the lesion), subarachnoid hemorrhage, spontaneous thrombosis with or without associated peripheral cerebrovascular incidents or even rupture. Rupture can be fatal, but in most cases it will cause a vertebro-venous fistula that may or may not be clinically significant. 

The advent of newer and highly advanced imaging modalities such as CT, Computed Tomography angiography (CTA) with 3D digital reconstruction, and MRI/MRA has greatly enhanced the ability to diagnose such lesions preoperatively; CT provides unique information about the size and configuration of the transverse foramina. CT angiography of the vertebral arteries suffers from the overlap of the bony structures and the time-consuming postprocessing bone removal. Contrast MR angiography with 3D FLASH sequence is the method of choice for the evaluation of the vertebral arteries. Digital subtraction angiography (DSA) depicts the resulting intraluminal compromise that may reveal some typical, but not specific, findings. The same is true for noninvasive angiographic techniques such as time-of-flight MRA and CTA, which have shown accurate results compared with DSA, but also allow the visualization of the vessel wall hematoma, confirming the diagnosis [3].

The majority of asymptomatic pseudoaneurysms are benign and follow-up imaging can be sufficient. Symptomatic lesions, however, will usually require treatment. Nowadays this is usually endovasular, either by occlusion or exclusion techniques, including diverters, although the application of the latter in the treatment of pseudoaneurysms remains to be explored further. Treatment, however, has to be tailored to the individual patient and is not risk-free; wide-necked giant aneurysms represent a limitation of the coiling procedures, due to unacceptable risk of coil migration. In such cases, combined treatment with coils and stents is the procedure of choice [4]. In our case, the aneurysm could have been packed with coils, but there was a high and predictable risk of partially occluding the vertebral artery. That risk was additional to that carried by coil migration owing to the wide neck of this aneurysm. Since our experience with stents was only limited, we proceeded with sacrifice of the affected artery. Primary surgical repair of vertebral artery aneurysms is notoriously difficult and should be considered in unstable patients with active hemorrhage and in the rare cases when the contralateral vertebral artery is hypoplastic or absent. 

In our case, we presume that the pseudoaneurysm was present for many years before becoming symptomatic. This is supported by the bony erosion demonstrated by 3D CT reconstruction. Chiropractic manipulation must have caused enlargement of the lesion which in turn caused cervical root impingement. Although the natural history of vertebral artery pseudoaneurysms is not well understood, this case supports a less minacious portrayal of vertebral artery aneurysms. It is possible that asymptomatic pseudoaneurysms are benign; whereas symptomatic ones are ominous lesions that will usually require treatment. 

 The responsibility lies upon the physician to utilize all the available imaging modalities to ensure the correct diagnosis and provide treatment by the least invasive and safest way. Every MRI should mandatorily use appropriate windows for ailments considered in the differential diagnosis; nevertheless the poor discriminatory value of MRI for vascular lesions only highlights the indispensability of MRA. The advent of newer and highly advanced imaging technologies such as CT, CT angiography with 3D digital reconstruction, and MRI/MRA has greatly enhanced the ability to diagnose such lesions preoperatively and should not be considered superfluous.

## Figures and Tables

**Figure 3:**
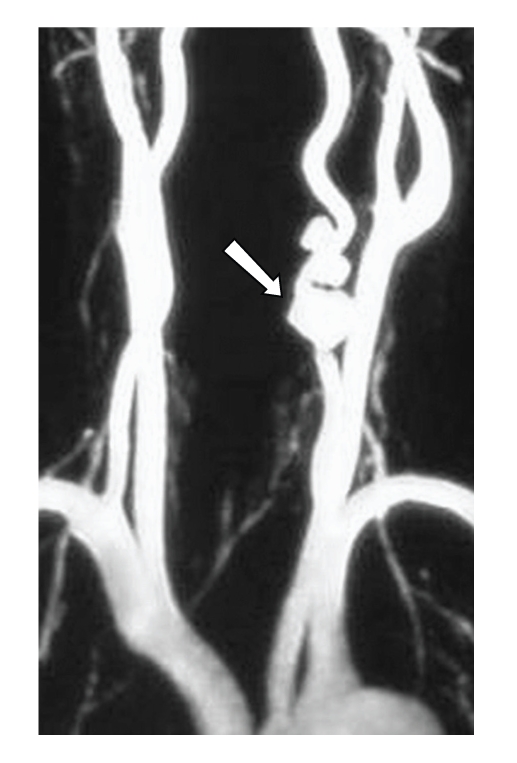
Digital Subtraction Angiography; arrow points the aneurysm.
